# Poly[diaqua(μ_2_-oxalato-κ^4^ *O* ^1^,*O* ^2^:*O* ^1′^,*O* ^2′^)(μ_2_-pyrazine-2-carboxyl­ato-κ^3^ *N* ^1^,*O*:*O*′)cerium(III)]

**DOI:** 10.1107/S1600536808029164

**Published:** 2008-09-20

**Authors:** Yuanlan Wang, Chongchen Wang

**Affiliations:** a Research Institute of Applied Chemistry, Central South University of Forestry and Technology, 412006 Zhuzhou, Hunan, People’s Republic of China; b School of Environmental and Energy Engineering, Beijing University of Civil Engineering and Architecture, 100044 Beijing, People’s Republic of China

## Abstract

In the hydro­thermally synthesized title compound, [Ce(C_5_H_3_N_2_O_2_)(C_2_O_4_)(H_2_O)_2_]_*n*_, the Ce^III^ ion is coordinated by four O atoms from two different oxalate ligands, three O atoms from two symmetry-related pyrazine-2-carboxyl­ate ligands, two O atoms from two water melecules and one N atom from a pyrazine-2-carboxyl­ate ligand in a distorted bicapped square-anti­prismatic coordination geometry. The oxalate and pyrazine-2-carboxyl­ate ligands bridge the Ce^III^ ions, forming a two-dimensional structure. In addition, inter­molecular O—H⋯O and O—H⋯N hydrogen bonds connect the two-dimensional structure into a three-dimensional network.

## Related literature

For background information, see: Eliseeva *et al.* (2004 ▶); Wang *et al.* (2007 ▶); Zou *et al.* (1999 ▶); Zheng *et al.* (2002 ▶). 
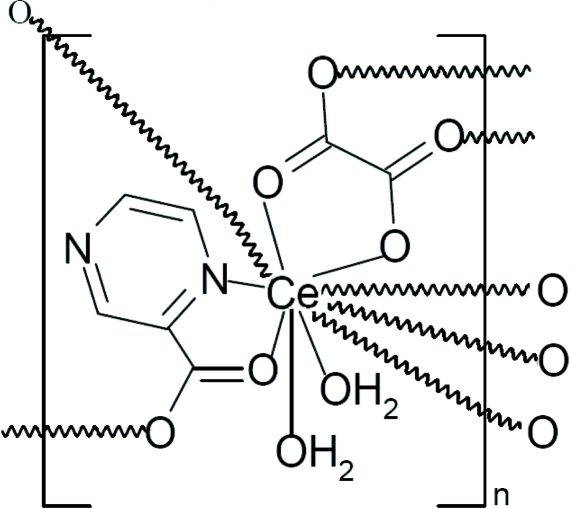


## Experimental

### 

#### Crystal data

[Ce(C_5_H_3_N_2_O_2_)(C_2_O_4_)(H_2_O)_2_]
*M* *_r_* = 387.27
Triclinic, 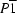

*a* = 8.0298 (7) Å
*b* = 8.7161 (9) Å
*c* = 8.8201 (9) Å
α = 115.514 (2)°
β = 101.747 (1)°
γ = 95.999 (1)°
*V* = 532.38 (9) Å^3^
*Z* = 2
Mo *K*α radiation
μ = 4.31 mm^−1^
*T* = 298 (2) K
0.24 × 0.15 × 0.10 mm

#### Data collection

Bruker SMART CCD area-detector diffractometer
Absorption correction: multi-scan (*SADABS*; Sheldrick, 1996 ▶) *T*_min_ = 0.424, *T*_max_ = 0.672
2790 measured reflections
1858 independent reflections
1760 reflections with *I* > 2σ(*I*)
*R*_int_ = 0.013

#### Refinement

*R*[*F* ^2^ > 2σ(*F* ^2^)] = 0.019
*wR*(*F* ^2^) = 0.049
*S* = 1.09
1858 reflections
163 parameters
H-atom parameters constrained
Δρ_max_ = 0.68 e Å^−3^
Δρ_min_ = −0.94 e Å^−3^

### 

Data collection: *SMART* (Bruker, 1996 ▶); cell refinement: *SAINT* (Bruker, 1996 ▶); data reduction: *SAINT*; program(s) used to solve structure: *SHELXS97* (Sheldrick, 2008 ▶); program(s) used to refine structure: *SHELXL97* (Sheldrick, 2008 ▶); molecular graphics: *SHELXTL* (Sheldrick, 2008 ▶); software used to prepare material for publication: *SHELXTL*.

## Supplementary Material

Crystal structure: contains datablocks global, I. DOI: 10.1107/S1600536808029164/lh2687sup1.cif

Structure factors: contains datablocks I. DOI: 10.1107/S1600536808029164/lh2687Isup2.hkl

Additional supplementary materials:  crystallographic information; 3D view; checkCIF report

## Figures and Tables

**Table 1:** Selected bond lengths (Å)

Ce1—O8	2.506 (2)
Ce1—O4^i^	2.521 (2)
Ce1—O5	2.530 (2)
Ce1—O3	2.538 (2)
Ce1—O6^ii^	2.540 (2)
Ce1—O1	2.578 (2)
Ce1—O7	2.595 (3)
Ce1—O1^iii^	2.614 (2)
Ce1—N1	2.815 (3)
Ce1—O2^iii^	2.897 (3)

Symmetry codes: (i) 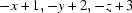; (ii) 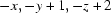; (iii) 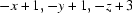.

**Table 2:** Hydrogen-bond geometry (Å, °)

*D*—H⋯*A*	*D*—H	H⋯*A*	*D*⋯*A*	*D*—H⋯*A*
O7—H7*A*⋯O5^iv^	0.85	2.10	2.836 (4)	145
O7—H7*B*⋯O2^v^	0.85	1.94	2.738 (4)	156
O8—H8*A*⋯N2^vi^	0.85	1.96	2.799 (4)	169
O8—H8*B*⋯O3^iii^	0.85	2.05	2.873 (3)	163

Symmetry codes: (iii) 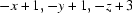; (iv) 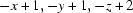; (v) 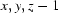; (vi) 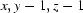.

**Fig. 1.**
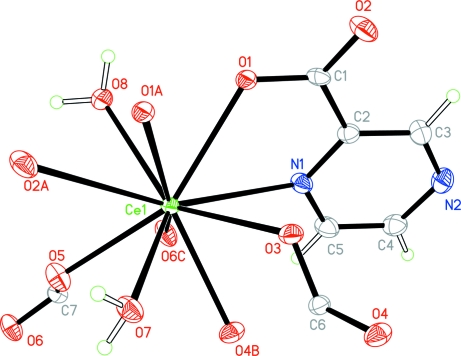
The asymmetric unit of the title compound with symmetry related atoms included to show the coordination environment of Ce1. Displacement ellipsoids are drawn at the 40% probability level [Symmetry codes: (A) -x + 1, -y + 2, -z + 3, (B) -x+1, -y+2, -z+3, (C) -x, -y+1, -z+2].

**Fig. 2.**
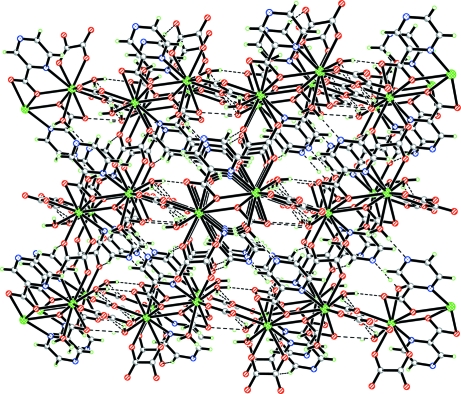
Part of the crystal structure showing hydrogen bonds as dashed lines.

## supplementary crystallographic information

### Comment

Rare metal coordination polymers of one-, two- and three-dimensional extended frameworks are an attractive research area because of the diverse structures available (Zheng, 2002; Eliseeva *et al.*, 2004). Pyrazine-2,3-dicaboxylic acid is a good ligand with a versatitle coordination mode, which is widely used in self-assembled polymeric coordination synthesis (Zou *et al.*, 1999; Wang *et al.*, 2007). The title compound, [Ce(C_5_H_3_N_2_O_2_)(C_2_O_4_)(H_2_O)_2_]*_n_*, was obtained unintentionally as the harvested product of the hydrothermal reaction of pyrazine-2,3-dicaboxylic acid and Ce_2_(C_2_O_4_)_3_.10H_2_O. We report here the crystal structure of the title compound, a 2-D polymeric structure consisting of pyrazine-2-dicaboxylate and oxalate ligands.

The coordination environment of the Ce^III^ ion can be described as a distorted bicapped square-antiprism, in which the Ce^III^ ion is ten-coordinated by four oxygen atoms from two different oxalate ligands, three oxygen atoms from two different pyrazine -2-carboxylic acid ligands, two oxygen atoms from two water molecules, and one nitrogen atom from a pyrazine-2-carboxylate ligand, as shown in the Fig. 1. The oxalate ligands and pyrazine-2-carboxylate ligands bridge Ce^III^ ions to form a two-dimensional structure. The Ce—O bond lengths range from 2.506 (2) to 2.897 (3) Å. In the crystal structure, intermolecular O—H···O and O—H···N hydrogen bonds connect the two-dimensional structure into a three dimensional network.

### Experimental

Colorless block-shaped crystals of the title compound were obtained by a hydrothermal reaction of Ce_2_(C_2_O_4_)_3_.10H_2_O (0.10 mmol, 0.0710 g), pyrazine-2,3-dicarboxylic acid (0.10 mmol, 0.0168 g) and deionized water (15 ml) in a 23 ml teflon-lined reaction vesset at 423 K for 120 h followed by slow cooling to room temperature (yield 77% based on initial input of pyrazine-2,3-dicarboxylic acid).

### Refinement

H atoms were included in calculated positions and refined in a riding-model approximation with O—H = 0.85 Å, C—H = 0.93 Å and U_iso_(H) = 1.2U_eq_(C,O).

### Crystal data

**Table:**

[Ce(C_5_H_3_N_2_O_2_)(C_2_O_4_)(H_2_O)_2_]	*Z* = 2
*M**_r_* = 387.27	*F*(000) = 370
Triclinic, *P*1	*D*_x_ = 2.416 Mg m^−^^3^
Hall symbol: -P 1	Mo *K*α radiation, λ = 0.71073 Å
*a* = 8.0298 (7) Å	Cell parameters from 2791 reflections
*b* = 8.7161 (9) Å	θ = 2.7–28.5°
*c* = 8.8201 (9) Å	µ = 4.31 mm^−^^1^
α = 115.514 (2)°	*T* = 298 K
β = 101.747 (1)°	Block, colourless
γ = 95.999 (1)°	0.24 × 0.15 × 0.10 mm
*V* = 532.38 (9) Å^3^	

### Data collection

**Table:**

Bruker SMART CCD area-detector diffractometer	1858 independent reflections
Radiation source: fine-focus sealed tube	1760 reflections with *I* > 2σ(*I*)
graphite	*R*_int_ = 0.013
φ and ω scans	θ_max_ = 25.0°, θ_min_ = 2.7°
Absorption correction: multi-scan (SADABS; Sheldrick, 1996)	*h* = −6→9
*T*_min_ = 0.424, *T*_max_ = 0.672	*k* = −10→10
2790 measured reflections	*l* = −10→8

### Refinement

**Table:**

Refinement on *F*^2^	Primary atom site location: structure-invariant direct methods
Least-squares matrix: full	Secondary atom site location: difference Fourier map
*R*[*F*^2^ > 2σ(*F*^2^)] = 0.019	Hydrogen site location: inferred from neighbouring sites
*wR*(*F*^2^) = 0.049	H-atom parameters constrained
*S* = 1.09	*w* = 1/[σ^2^(*F*_o_^2^) + (0.0274*P*)^2^ + 0.3347*P*] where *P* = (*F*_o_^2^ + 2*F*_c_^2^)/3
1858 reflections	(Δ/σ)_max_ = 0.002
163 parameters	Δρ_max_ = 0.68 e Å^−^^3^
0 restraints	Δρ_min_ = −0.95 e Å^−^^3^

### Special details

**Table:**

Geometry. All e.s.d.'s (except the e.s.d. in the dihedral angle between two l.s. planes) are estimated using the full covariance matrix. The cell e.s.d.'s are taken into account individually in the estimation of e.s.d.'s in distances, angles and torsion angles; correlations between e.s.d.'s in cell parameters are only used when they are defined by crystal symmetry. An approximate (isotropic) treatment of cell e.s.d.'s is used for estimating e.s.d.'s involving l.s. planes.
Refinement. Refinement of *F*^2^ against ALL reflections. The weighted *R*-factor *wR* and goodness of fit *S* are based on *F*^2^, conventional *R*-factors *R* are based on *F*, with *F* set to zero for negative *F*^2^. The threshold expression of *F*^2^ > σ(*F*^2^) is used only for calculating *R*-factors(gt) *etc*. and is not relevant to the choice of reflections for refinement. *R*-factors based on *F*^2^ are statistically about twice as large as those based on *F*, and *R*- factors based on ALL data will be even larger.

### Fractional atomic coordinates and isotropic or equivalent isotropic displacement parameters (Å^2^)

**Table:**

	*x*	*y*	*z*	*U*_iso_*/*U*_eq_	
Ce1	0.33921 (2)	0.58558 (2)	1.32325 (2)	0.01398 (8)	
N1	0.1921 (4)	0.7628 (4)	1.5966 (4)	0.0211 (6)	
N2	0.1556 (4)	1.0173 (4)	1.9097 (4)	0.0265 (7)	
O1	0.3927 (3)	0.5389 (3)	1.5971 (3)	0.0193 (5)	
O2	0.4946 (3)	0.6763 (4)	1.8853 (3)	0.0287 (6)	
O3	0.5575 (3)	0.8498 (3)	1.5730 (3)	0.0214 (5)	
O4	0.6890 (3)	1.1263 (3)	1.6700 (3)	0.0224 (5)	
O5	0.2132 (3)	0.4771 (4)	0.9986 (3)	0.0257 (6)	
O6	−0.0157 (3)	0.4293 (4)	0.7792 (3)	0.0257 (6)	
O7	0.5757 (3)	0.6984 (3)	1.2121 (3)	0.0251 (6)	
H7A	0.6535	0.6401	1.1878	0.030*	
H7B	0.5255	0.7040	1.1205	0.030*	
O8	0.2021 (3)	0.2812 (3)	1.2493 (3)	0.0228 (5)	
H8A	0.1736	0.2001	1.1447	0.027*	
H8B	0.2566	0.2414	1.3126	0.027*	
C1	0.3960 (4)	0.6567 (4)	1.7474 (5)	0.0186 (7)	
C2	0.2763 (4)	0.7791 (4)	1.7518 (5)	0.0194 (7)	
C3	0.2585 (5)	0.9048 (5)	1.9071 (5)	0.0258 (8)	
H3	0.3192	0.9114	2.0122	0.031*	
C4	0.0669 (5)	0.9965 (5)	1.7544 (5)	0.0273 (8)	
H4	−0.0092	1.0689	1.7502	0.033*	
C5	0.0845 (5)	0.8704 (5)	1.5992 (5)	0.0264 (8)	
H5	0.0197	0.8603	1.4939	0.032*	
C6	0.5713 (4)	0.9932 (4)	1.5705 (4)	0.0159 (7)	
C7	0.0567 (4)	0.4734 (4)	0.9350 (4)	0.0187 (7)	

### Atomic displacement parameters (Å^2^)

**Table:**

	*U*^11^	*U*^22^	*U*^33^	*U*^12^	*U*^13^	*U*^23^
Ce1	0.01536 (12)	0.01406 (12)	0.01362 (12)	0.00340 (8)	0.00329 (8)	0.00769 (9)
N1	0.0232 (16)	0.0251 (16)	0.0198 (16)	0.0104 (13)	0.0082 (12)	0.0127 (13)
N2	0.0270 (17)	0.0240 (16)	0.0273 (18)	0.0080 (13)	0.0117 (14)	0.0084 (14)
O1	0.0259 (13)	0.0191 (12)	0.0190 (13)	0.0111 (10)	0.0106 (10)	0.0108 (10)
O2	0.0305 (15)	0.0407 (16)	0.0213 (14)	0.0144 (12)	0.0069 (11)	0.0185 (12)
O3	0.0258 (13)	0.0179 (12)	0.0206 (13)	0.0034 (10)	0.0015 (10)	0.0115 (10)
O4	0.0215 (13)	0.0180 (12)	0.0264 (14)	0.0034 (10)	0.0006 (11)	0.0120 (11)
O5	0.0162 (13)	0.0410 (16)	0.0198 (13)	0.0090 (11)	0.0044 (10)	0.0138 (12)
O6	0.0200 (13)	0.0389 (15)	0.0165 (13)	0.0077 (11)	0.0033 (10)	0.0118 (12)
O7	0.0266 (14)	0.0329 (15)	0.0230 (14)	0.0106 (11)	0.0128 (11)	0.0157 (12)
O8	0.0283 (14)	0.0169 (12)	0.0202 (13)	0.0021 (10)	0.0042 (11)	0.0077 (11)
C1	0.0210 (18)	0.0223 (18)	0.0204 (19)	0.0057 (14)	0.0096 (14)	0.0151 (15)
C2	0.0201 (18)	0.0201 (17)	0.0225 (18)	0.0069 (14)	0.0087 (14)	0.0120 (15)
C3	0.026 (2)	0.028 (2)	0.0213 (19)	0.0070 (16)	0.0066 (15)	0.0098 (16)
C4	0.030 (2)	0.027 (2)	0.032 (2)	0.0126 (16)	0.0133 (17)	0.0166 (17)
C5	0.030 (2)	0.032 (2)	0.026 (2)	0.0148 (17)	0.0102 (16)	0.0180 (17)
C6	0.0176 (17)	0.0146 (17)	0.0178 (17)	0.0049 (13)	0.0068 (14)	0.0085 (14)
C7	0.0201 (18)	0.0203 (18)	0.0183 (18)	0.0038 (14)	0.0060 (15)	0.0109 (15)

### Geometric parameters (Å, °)

**Table:**

Ce1—O8	2.506 (2)	O3—C6	1.254 (4)
Ce1—O4^i^	2.521 (2)	O4—C6	1.251 (4)
Ce1—O5	2.530 (2)	O4—Ce1^i^	2.521 (2)
Ce1—O3	2.538 (2)	O5—C7	1.259 (4)
Ce1—O6^ii^	2.540 (2)	O6—C7	1.244 (4)
Ce1—O1	2.578 (2)	O6—Ce1^ii^	2.540 (2)
Ce1—O7	2.595 (3)	O7—H7A	0.8500
Ce1—O1^iii^	2.614 (2)	O7—H7B	0.8500
Ce1—N1	2.815 (3)	O8—H8A	0.8500
Ce1—O2^iii^	2.897 (3)	O8—H8B	0.8500
N1—C5	1.337 (5)	C1—C2	1.502 (5)
N1—C2	1.337 (5)	C2—C3	1.384 (5)
N2—C4	1.333 (5)	C3—H3	0.9300
N2—C3	1.343 (5)	C4—C5	1.385 (5)
O1—C1	1.273 (4)	C4—H4	0.9300
O1—Ce1^iii^	2.614 (2)	C5—H5	0.9300
O2—C1	1.240 (4)	C6—C6^i^	1.564 (6)
O2—Ce1^iii^	2.897 (3)	C7—C7^ii^	1.554 (7)
			
O8—Ce1—O4^i^	149.92 (8)	O7—Ce1—O2^iii^	64.80 (8)
O8—Ce1—O5	82.87 (9)	O1^iii^—Ce1—O2^iii^	46.97 (7)
O4^i^—Ce1—O5	81.64 (8)	N1—Ce1—O2^iii^	157.52 (8)
O8—Ce1—O3	139.44 (8)	C5—N1—C2	116.2 (3)
O4^i^—Ce1—O3	64.55 (7)	C5—N1—Ce1	126.2 (2)
O5—Ce1—O3	136.12 (8)	C2—N1—Ce1	114.7 (2)
O8—Ce1—O6^ii^	76.91 (9)	C4—N2—C3	116.2 (3)
O4^i^—Ce1—O6^ii^	73.14 (8)	C1—O1—Ce1	122.9 (2)
O5—Ce1—O6^ii^	63.80 (8)	C1—O1—Ce1^iii^	100.9 (2)
O3—Ce1—O6^ii^	124.88 (8)	Ce1—O1—Ce1^iii^	119.65 (9)
O8—Ce1—O1	68.68 (8)	C1—O2—Ce1^iii^	88.3 (2)
O4^i^—Ce1—O1	123.57 (8)	C6—O3—Ce1	119.4 (2)
O5—Ce1—O1	151.55 (9)	C6—O4—Ce1^i^	120.1 (2)
O3—Ce1—O1	71.80 (8)	C7—O5—Ce1	121.5 (2)
O6^ii^—Ce1—O1	107.92 (8)	C7—O6—Ce1^ii^	121.0 (2)
O8—Ce1—O7	130.61 (8)	Ce1—O7—H7A	117.4
O4^i^—Ce1—O7	67.79 (8)	Ce1—O7—H7B	108.6
O5—Ce1—O7	72.58 (8)	H7A—O7—H7B	107.4
O3—Ce1—O7	69.25 (8)	Ce1—O8—H8A	120.9
O6^ii^—Ce1—O7	124.39 (8)	Ce1—O8—H8B	114.4
O1—Ce1—O7	126.31 (8)	H8A—O8—H8B	106.6
O8—Ce1—O1^iii^	77.10 (8)	O2—C1—O1	123.3 (3)
O4^i^—Ce1—O1^iii^	132.90 (8)	O2—C1—C2	120.1 (3)
O5—Ce1—O1^iii^	114.25 (8)	O1—C1—C2	116.6 (3)
O3—Ce1—O1^iii^	75.86 (7)	N1—C2—C3	122.0 (3)
O6^ii^—Ce1—O1^iii^	153.96 (9)	N1—C2—C1	115.8 (3)
O1—Ce1—O1^iii^	60.35 (9)	C3—C2—C1	122.2 (3)
O7—Ce1—O1^iii^	75.23 (8)	N2—C3—C2	121.6 (4)
O8—Ce1—N1	97.71 (9)	N2—C3—H3	119.2
O4^i^—Ce1—N1	72.66 (9)	C2—C3—H3	119.2
O5—Ce1—N1	128.42 (8)	N2—C4—C5	122.1 (3)
O3—Ce1—N1	68.56 (9)	N2—C4—H4	119.0
O6^ii^—Ce1—N1	66.20 (8)	C5—C4—H4	119.0
O1—Ce1—N1	58.85 (8)	N1—C5—C4	121.8 (3)
O7—Ce1—N1	131.17 (9)	N1—C5—H5	119.1
O1^iii^—Ce1—N1	116.07 (8)	C4—C5—H5	119.1
O8—Ce1—O2^iii^	66.33 (8)	O4—C6—O3	126.0 (3)
O4^i^—Ce1—O2^iii^	129.05 (8)	O4—C6—C6^i^	117.0 (3)
O5—Ce1—O2^iii^	67.54 (8)	O3—C6—C6^i^	117.0 (4)
O3—Ce1—O2^iii^	112.59 (8)	O6—C7—O5	126.5 (3)
O6^ii^—Ce1—O2^iii^	121.33 (8)	O6—C7—C7^ii^	117.4 (4)
O1—Ce1—O2^iii^	99.44 (7)	O5—C7—C7^ii^	116.1 (4)
			
O8—Ce1—N1—C5	−117.5 (3)	O1^iii^—Ce1—O3—C6	142.2 (3)
O4^i^—Ce1—N1—C5	33.2 (3)	N1—Ce1—O3—C6	−91.9 (2)
O5—Ce1—N1—C5	−30.5 (4)	O2^iii^—Ce1—O3—C6	112.3 (2)
O3—Ce1—N1—C5	102.1 (3)	O8—Ce1—O5—C7	82.8 (3)
O6^ii^—Ce1—N1—C5	−45.6 (3)	O4^i^—Ce1—O5—C7	−71.3 (3)
O1—Ce1—N1—C5	−176.8 (3)	O3—Ce1—O5—C7	−110.1 (3)
O7—Ce1—N1—C5	70.2 (3)	O6^ii^—Ce1—O5—C7	3.9 (3)
O1^iii^—Ce1—N1—C5	163.1 (3)	O1—Ce1—O5—C7	83.1 (3)
O2^iii^—Ce1—N1—C5	−160.4 (3)	O7—Ce1—O5—C7	−140.6 (3)
O8—Ce1—N1—C2	82.8 (3)	O1^iii^—Ce1—O5—C7	155.2 (3)
O4^i^—Ce1—N1—C2	−126.4 (3)	N1—Ce1—O5—C7	−11.4 (3)
O5—Ce1—N1—C2	169.8 (2)	O2^iii^—Ce1—O5—C7	150.1 (3)
O3—Ce1—N1—C2	−57.6 (2)	Ce1^iii^—O2—C1—O1	6.9 (3)
O6^ii^—Ce1—N1—C2	154.8 (3)	Ce1^iii^—O2—C1—C2	−171.1 (3)
O1—Ce1—N1—C2	23.5 (2)	Ce1—O1—C1—O2	−144.3 (3)
O7—Ce1—N1—C2	−89.5 (3)	Ce1^iii^—O1—C1—O2	−7.8 (4)
O1^iii^—Ce1—N1—C2	3.4 (3)	Ce1—O1—C1—C2	33.8 (4)
O2^iii^—Ce1—N1—C2	39.9 (4)	Ce1^iii^—O1—C1—C2	170.3 (3)
O8—Ce1—O1—C1	−144.1 (3)	C5—N1—C2—C3	−2.5 (5)
O4^i^—Ce1—O1—C1	4.7 (3)	Ce1—N1—C2—C3	159.3 (3)
O5—Ce1—O1—C1	−144.4 (2)	C5—N1—C2—C1	179.6 (3)
O3—Ce1—O1—C1	45.2 (2)	Ce1—N1—C2—C1	−18.6 (4)
O6^ii^—Ce1—O1—C1	−76.6 (3)	O2—C1—C2—N1	171.3 (3)
O7—Ce1—O1—C1	90.4 (3)	O1—C1—C2—N1	−6.8 (5)
O1^iii^—Ce1—O1—C1	129.0 (3)	O2—C1—C2—C3	−6.6 (5)
N1—Ce1—O1—C1	−30.3 (2)	O1—C1—C2—C3	175.3 (3)
O2^iii^—Ce1—O1—C1	156.0 (3)	C4—N2—C3—C2	2.5 (6)
O8—Ce1—O1—Ce1^iii^	86.94 (11)	N1—C2—C3—N2	−0.1 (6)
O4^i^—Ce1—O1—Ce1^iii^	−124.21 (10)	C1—C2—C3—N2	177.7 (3)
O5—Ce1—O1—Ce1^iii^	86.64 (18)	C3—N2—C4—C5	−2.4 (6)
O3—Ce1—O1—Ce1^iii^	−83.76 (11)	C2—N1—C5—C4	2.6 (6)
O6^ii^—Ce1—O1—Ce1^iii^	154.49 (10)	Ce1—N1—C5—C4	−156.8 (3)
O7—Ce1—O1—Ce1^iii^	−38.53 (14)	N2—C4—C5—N1	−0.2 (6)
O1^iii^—Ce1—O1—Ce1^iii^	0.0	Ce1^i^—O4—C6—O3	−168.9 (3)
N1—Ce1—O1—Ce1^iii^	−159.23 (14)	Ce1^i^—O4—C6—C6^i^	11.3 (5)
O2^iii^—Ce1—O1—Ce1^iii^	27.05 (11)	Ce1—O3—C6—O4	−169.0 (3)
O8—Ce1—O3—C6	−168.2 (2)	Ce1—O3—C6—C6^i^	10.8 (5)
O4^i^—Ce1—O3—C6	−11.6 (2)	Ce1^ii^—O6—C7—O5	176.2 (3)
O5—Ce1—O3—C6	31.8 (3)	Ce1^ii^—O6—C7—C7^ii^	−2.7 (5)
O6^ii^—Ce1—O3—C6	−55.2 (3)	Ce1—O5—C7—O6	176.9 (3)
O1—Ce1—O3—C6	−154.8 (3)	Ce1—O5—C7—C7^ii^	−4.2 (5)
O7—Ce1—O3—C6	62.9 (2)		

Symmetry codes: (i) −*x*+1, −*y*+2, −*z*+3; (ii) −*x*, −*y*+1, −*z*+2; (iii) −*x*+1, −*y*+1, −*z*+3.

### Hydrogen-bond geometry (Å, °)

**Table:**

*D*—H···*A*	*D*—H	H···*A*	*D*···*A*	*D*—H···*A*
O7—H7A···O5^iv^	0.85	2.10	2.836 (4)	145.
O7—H7B···O2^v^	0.85	1.94	2.738 (4)	156.
O8—H8A···N2^vi^	0.85	1.96	2.799 (4)	169.
O8—H8B···O3^iii^	0.85	2.05	2.873 (3)	163.

Symmetry codes: (iv) −*x*+1, −*y*+1, −*z*+2; (v) *x*, *y*, *z*−1; (vi) *x*, *y*−1, *z*−1; (iii) −*x*+1, −*y*+1, −*z*+3.
